# N,N-Dimethlyacetamide Prevents the High-Fat Diet-Induced Increase in Body Weight

**DOI:** 10.3389/fphar.2019.01274

**Published:** 2019-10-30

**Authors:** Indranil Bhattacharya, Chafik Ghayor, Ana Pérez Dominguez, Franz E. Weber

**Affiliations:** ^1^Oral Biotechnology and Bioengineering, Department of Cranio-Maxillofacial and Oral Surgery, Center for Dental Medicine, University of Zurich, Zurich, Switzerland; ^2^Centre for Applied Biotechnology and Molecular Medicine, University of Zurich, Zurich, Switzerland; ^3^Zurich Centre for Integrative Human Physiology, University of Zurich, Zurich, Switzerland

**Keywords:** N, N-dimethyacetamide, high-fat diet, body weight, adiposity, adipogenesis, fat

## Abstract

Increased body weight caused by visceral fat accumulation is on the rise and is reaching epidemic proportions worldwide. Hence, means and ways to tackle the problem of increased adiposity is of utmost importance. In this work, we report the effect of a water-soluble small molecule N,N-Dimethlyacetamide (DMA) on weight gain and adiposity *in vitro* and *in vivo*. To monitor the *in vitro* effect of DMA on adipogenesis, 3T3-L1 preadipocytes and pluripotent C2C12 cells were differentiated to adipocytes in the presence of DMA (5 mM and 10 mM). Oil red O staining and reverse transcriptase polymerase chain reaction (RT-PCR) were performed to evaluate the differentiation to adipocytes. To study the *in vivo* effect of DMA on body weight, experiments were done with C57BL/6J male mice (6 weeks old). The mice were randomly assigned to receive either high-fat diet (HFD; 45% fat) or a normal diet (7% fat) and were either intraperitoneally injected with DMA or phosphate-buffered saline (PBS) once a week for 20 weeks. Glucose tolerance test was performed on living mice. Post-experiment, the epididymal and subcutaneous adipose tissue were excised from the sacrificed animal, and histology, RT-PCR and plasma triglyceride assay were performed. DMA had no inhibitory effect on adipocyte differentiation when applied only once. However, sustained treatment with DMA inhibited the adipocyte differentiation in both 3T3-L1 and C2C12 cells, and significantly lowered the expression of adipocyte markers, in particular, fatty acid-binding protein 4 (fabp4). Under HFD, C57BL/6J mice treated with DMA had lower body weight compared with PBS treatment. Moreover, the HFD-induced higher body weight was controlled when the mice were switched from PBS to DMA treatment. Further, the HFD-mediated adipocyte hypertrophy from epididymal and subcutaneous adipose tissue was significantly reduced with DMA treatment. Interestingly, the glucose clearance and triglyceride levels in the plasma were improved in mice when DMA treatment was initiated early. Taken together, our results show that DMA exhibits a clear potential to prevent weight gain and restricts adiposity in response to high-fat feeding.

## Introduction

Increased body weight in both children and in adults due to high visceral fat deposition is a major health concern. Over the last 40 years, the global prevalence of overweight and abdominal obesity has risen in both developed and developing countries, mainly in the cities (Ng et al., 2014). A detailed policy paper forecasting life expectancy suggest that if the present trend of overweight/obesity continues then increased body mass index (BMI) would be the topmost contributor for reduced life expectancy (Foreman et al., 2018). It is now well established that obesity/overweight is associated with major non-communicable metabolic diseases like diabetes, cardiovascular diseases and certain forms of cancer. Moreover, the bone health is often deteriorated in individuals with increased fat mass (Shapses et al., 2017). The prevalence of vertebral fractures are higher in individuals with obesity (Rudman et al., 2019) and the occurrence of osteoporosis, particularly in postmenopausal women, is associated with increased body weight (Hsu et al., 2006). High-fat diet-induced body weight gain enhance bone resorption and elevate osteoclasts in bone marrow (Cao et al., 2010). Indeed lifestyle changes such as calorie restriction along with exercise has been reported to reduce fat mass, improve bone health and other cardio-metabolic parameters (Villareal et al., 2011; Styner et al., 2017: Oh et al., 2018).

Work from our group have shown that small molecules like NMP (N-methyl pyrrolidone) and DMA (*N*,*N*-Dimethylaceteamide) which are used for drug delivery are also inherently bioactive (Gjoksi et al., 2016; Ghayor et al., 2017). We recently showed that DMA, a water-miscible solvent and a FDA approved excipient is epigenetically active and inhibits osteoclastogenesis and inflammation (Ghayor et al., 2017). Others have also shown that DMA reduces inflammation-induced preterm birth (Gorasiya et al., 2018), which is mediated by inhibiting the nuclear factor-κB pathway (Pekson et al., 2016).

Using ovariectomized rats where estradiol depletion increases body weight, we have demonstrated that DMA blocks the body weight increase (Ghayor et al., 2017). Since ovariectomy is an artificial model leading to weight gain, hence in this work we asked the question if the bioactive small molecule DMA could control or prevent the weight gain induced by high-fat diet feeding.

## Materials and Methods

### Cell Culture and Differentiation

3T3-L1 cells were cultured and differentiated at 37°C and 5% CO_2_, as previously described (Bhattacharya et al., 2015). Briefly, 3T3-L1 cells purchased from American Type Culture Collection (ATCC, Manassas, VA, USA) were grown to confluence in Dulbecco’s modified Eagle’s medium (DMEM+GlutaMax-1; GIBCO, Grand Island, USA) with 10% new born calf serum (NBCS, Gibco, New Zealand) and antibiotics (100 U/ml penicillin G and 100 mg/ml streptomycin). 2 days post-confluence, the induction media (IM) comprising of 3-isobutyl-1 methylxanthine (0.5 mM), dexamethasone (0.25 μM), insulin (10 μg/ml) and rosiglitazone (2 μM) in DMEM with 10% fetal bovine serum (FBS) was applied. The components of IM are also referred as MDIR in the manuscript. The IM was replaced after 2 days with the differentiation media (DM) comprising of DMEM with 10% FBS and insulin (1 μg/ml). Thereafter throughout the differentiation, the DM was changed every 2nd day.

N, N-Dimethylacetamide (DMA) anhydrous with 99.8% purity (Sigma-Aldrich Chemie GmbH, St. Gallen, Switzerland) was used for the experiments. DMA at 5mM and 10mM was added for these durations: a) Throughout: from day 0 to day 10; b) Post-Induction: from day 2 to day 10; c) Only once with IM: day 0 to day 2 and d) Post-differentiation: from day 10 to day 20.

C2C12 cells were cultured and differentiated at 37°C and 5% CO_2_, as previously described (Qi et al., 2017). C2C12 cells (ATCC, Manassas, VA, USA) were cultured in DMEM media supplemented with 10% FBS and antibiotics (100 U/ml penicillin G and 100 mg/ml streptomycin). Once confluent, cells were treated in a similar manner like 3T3-L1 cells. DMA (5 and 10 mM) was added throughout the differentiation period.

### Oil Red O Staining

Staining with Oil red O was done as previously described (Gjoksi et al., 2016). Briefly, cells fixed with 10% formalin were washed with 60% isopropanol and were subsequently stained with 0.5% Oil red O for 10 min. The non-specific staining was washed with water. For quantification, 100% isopropanol was applied to the stained cells and the stain was eluted, and the absorbance was measured at 500 nm.

### Mice

Four weeks old, male C57BL/6J mice were procured from Charles River Laboratory (Sulzfeld, Germany). Three mice per cage were housed at the institutional animal facilities (University Hospital Zurich, Switzerland) with a 12-h light/dark cycle. At 6 weeks of age, the mice were randomly given either standard chow (7% calories from fat; Kliba Nafag, Kaiseraugust, Switzerland) or high-fat diet (HFD; 45% calories from fat; Research Diets, Inc. New Brunswick, NJ, USA) for 20 weeks and water ad libitum. The mice fed with standard chow or HFD were either intraperitoneally injected with Dulbecco’s phosphate buffered saline (PBS; Gibco, Thermo Fisher Scientific, MA, USA) or with DMA (99.8% purity, Sigma-Aldrich Chemie, GmbH, Switzerland) once a week. DMA injected was 1/4^th^ the dose of LD_50_ (ml/kg) as previously described (Bartsch et al., 1976). Before sacrificing, mice were weighed and put to sleep by exposing to CO_2_ and sacrifice was done by cervical dislocation. The local authorities (Kantonales Veterinaeramt of Zurich, Switzerland) approved the animal experiments.

#### Reverse Transcription Quantitative Polymerase Chain Reaction Analysis

RNA was extracted by scrapping the cells from the plate, as described earlier (Bhattacharya et al., 2015). RNA from epididymal adipose tissue was extracted after homogenizing the adipose tissue using homogenizer (Qiagen, Hombrechtikon, Switzerland). Briefly, RNeasy^®^ lipid tissue kit was used to extract RNA from adipocytes as per the protocol of the manufacturer (Qiagen, Hombrechtikon, Switzerland). RNA quantity was determined using Nanodrop 2000 Spectrophotometer. Reverse transcription of 1 µg RNA was perfomed using iScript™ Reverse Transcription Supermix according to manufacturer’s recommendations (BioRad, Hercules, CA, USA). Polymerase chain reaction was performed with each sample in duplicates using Bio-Rad CFX96 Real-Time System and iQ™ SYBR^®^ Green Supermix (BioRad, Hercules, CA, USA) using specific primers. Gene expression was normalized to the reference gene ribosomal protein s18 (rps18) using the comparative C_T_ method.

### Primers

The primers that were used in this study were commercially available and was purchased from Qiagen. All the primers were specific to mouse (**Table 1**).

**Table 1:** List of primers used in this study.

Gene symbol	Product	Cat. no.:
Rps18	ribosomal protein S18	QT00324940
Fabp4	fatty acid binding protein 4	QT00091532
Pparg	peroxisome proliferator activated receptor gamma	QT00100296
Cebpa	CCAAT/enhancer binding protein (C/EBP), alpha	QT00395010
Adipoq	Adiponectin	QT01169343
Scd1	stearoyl-Coenzyme A desaturase 1	QT00291151
Il6	interleukin 6	QT00098875

### Glucose Tolerance Test

The glucose tolerance test was done as described earlier (Mundy et al., 2007). Briefly, the mice were starved overnight with access to water and starving blood glucose was measured. Glucose (2mg/g) body weight was injected intraperitoneally and blood glucose levels were measured at 15, 30, 60, 90, 120 min. The starving blood glucose (mmol/L) was measured using Accu-Chek Aviva Blood glucose meter (Roche Diabetes Care GmbH, Mannheim, Germany).

### Histology

The paraffin sections of the epididymal and subcutaneous adipose tissue, and liver samples were processed and stained with hematoxylin and eosin. The sections were visualized by using the Olympus CKX53 microscope and the images were captured using the software Olympus CellSense Entry 1.16.

### Triglyceride Assay

The levels of triglyceride in the plasma were measured using the commercial available triglyceride assay kit (ab65336) from Abcam (Cambridge, UK). All the steps were followed as per the instructions in the manual.

### Statistical Analysis

Data are expressed as means ± standard error of the mean (SEM). Comparisons between groups were analyzed by two-tailed Student’s t-test. Differences were considered statistically significant at values of P < 0.05. The sample size was determined by power analysis using the online G*Power tool (Heinrich Heine Universität Düsseldorf, Germany).

All statistical analysis was performed using GraphPad Prism 5.04 program for Windows (GraphPad software, San Diego, CA, USA).

## Results

### DMA Inhibits Adipogenesis

**Figure 1:**
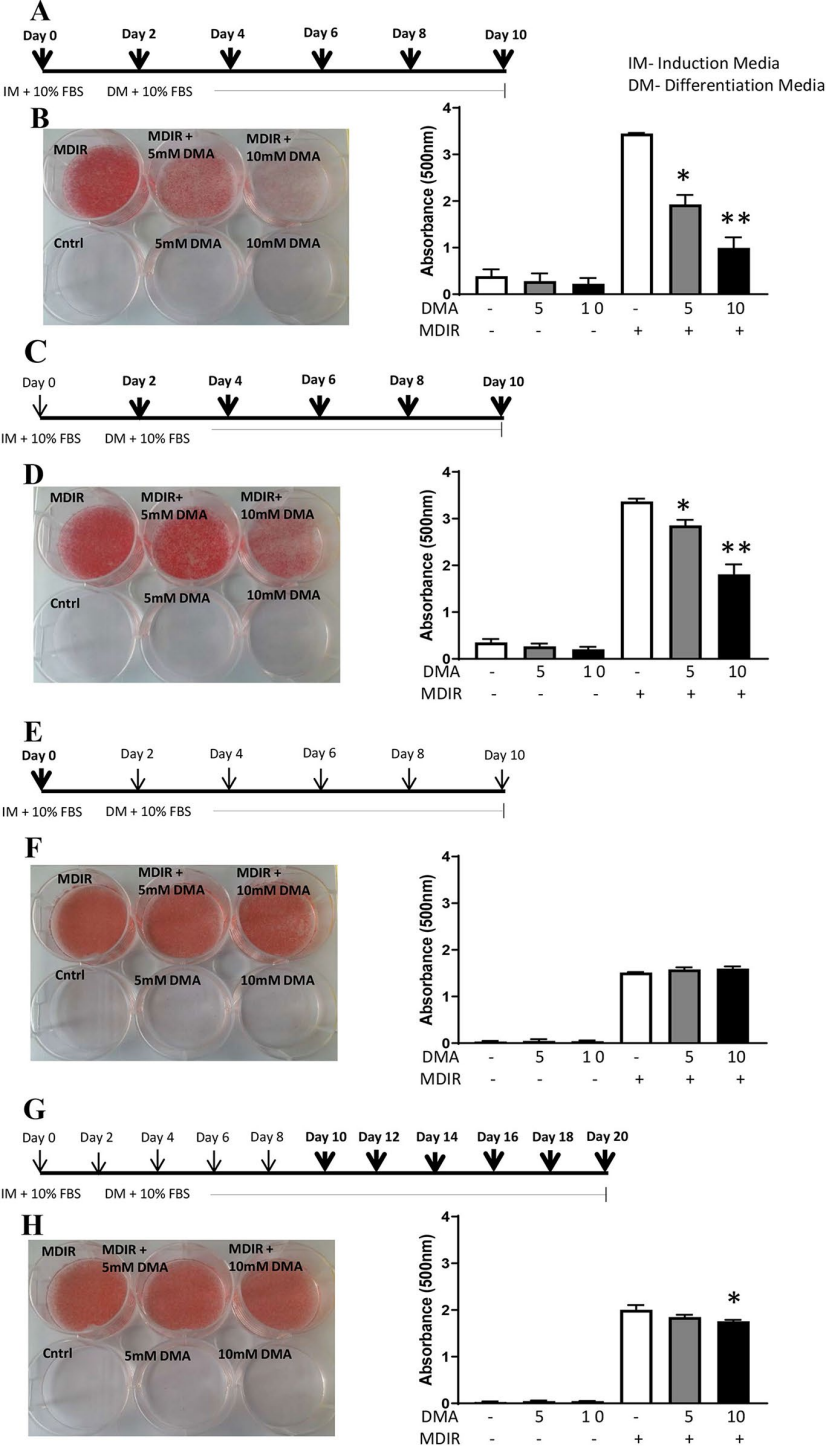
3T3-L1 cells were differentiated to adipocytes in the presence or absence of DMA. **(A**, **B)** DMA was applied throughout with the induction media (IM) and differentiation media (DM) from day 0 to day 10. **(C**, **D)** DMA was applied throughout the differentiation media (DM) from day 2 to day 10. **(E**, **F)** DMA was applied only once with the induction media (IM) on day 0. **(G**, **H)** DMA was applied to the differentiated 3T3-L1 adipocytes from day 10 to day 20. The sketch indicates the period of differentiation and bold arrows indicate the points of DMA treatment. MDIR (3-isobutyl-1 methylxanthine, dexamethasone, insulin and rosiglitazone) was added in absence or presence of 5mM or 10mM DMA. Controls were without MDIR and without or with DMA. The red staining indicates the staining by Oil-red O. The staining was eluted, absorbance was measured and represented graphically. *p ≤ 0.05 vs. MDIR; **p ≤ 0.05 vs. MDIR with 5 mM DMA, n = 3 independent experiments.

The 3T3-L1 preadipocytes were differentiated to adipocytes in the presence of the differential cocktail (MDIR). When 5 mM or 10 mM DMA was given throughout the differentiation process from day 0 to day 10 (**Figure 1A**), the differentiation was inhibited in a concentration-dependent manner (**Figure 1B**). DMA 5 mM and 10 mM inhibited differentiation by 1.8- and 3.8-fold, respectively. In the absence of MDIR, the cells did not differentiate.

When 5 mM or 10 mM DMA was applied post-induction of differentiation from Day 2 to Day 10 (**Figure 1C**), the differentiation was inhibited but by a lesser degree. DMA 5 mM and 10 mM inhibited the differentiation by 1.2- and 1.8-fold, respectively (**Figure 1D**). When DMA was applied only once during the induction of differentiation (**Figure 1E**), the differentiation was unaffected either by 5mM or 10mM DMA (**Figure 1F**). To examine if DMA had an effect on differentiated adipocytes, DMA applied from day 10 to day 20 (**Figure 1G**) caused only a marginal reduction in differentiation with 10mM DMA (**Figure 1H**), as determined by Oil Red O staining (1.1-fold; p = 0.01; **Figure 1H**).

### DMA Lowers the Gene Expression of Adipocyte Markers

**Figure 2:**
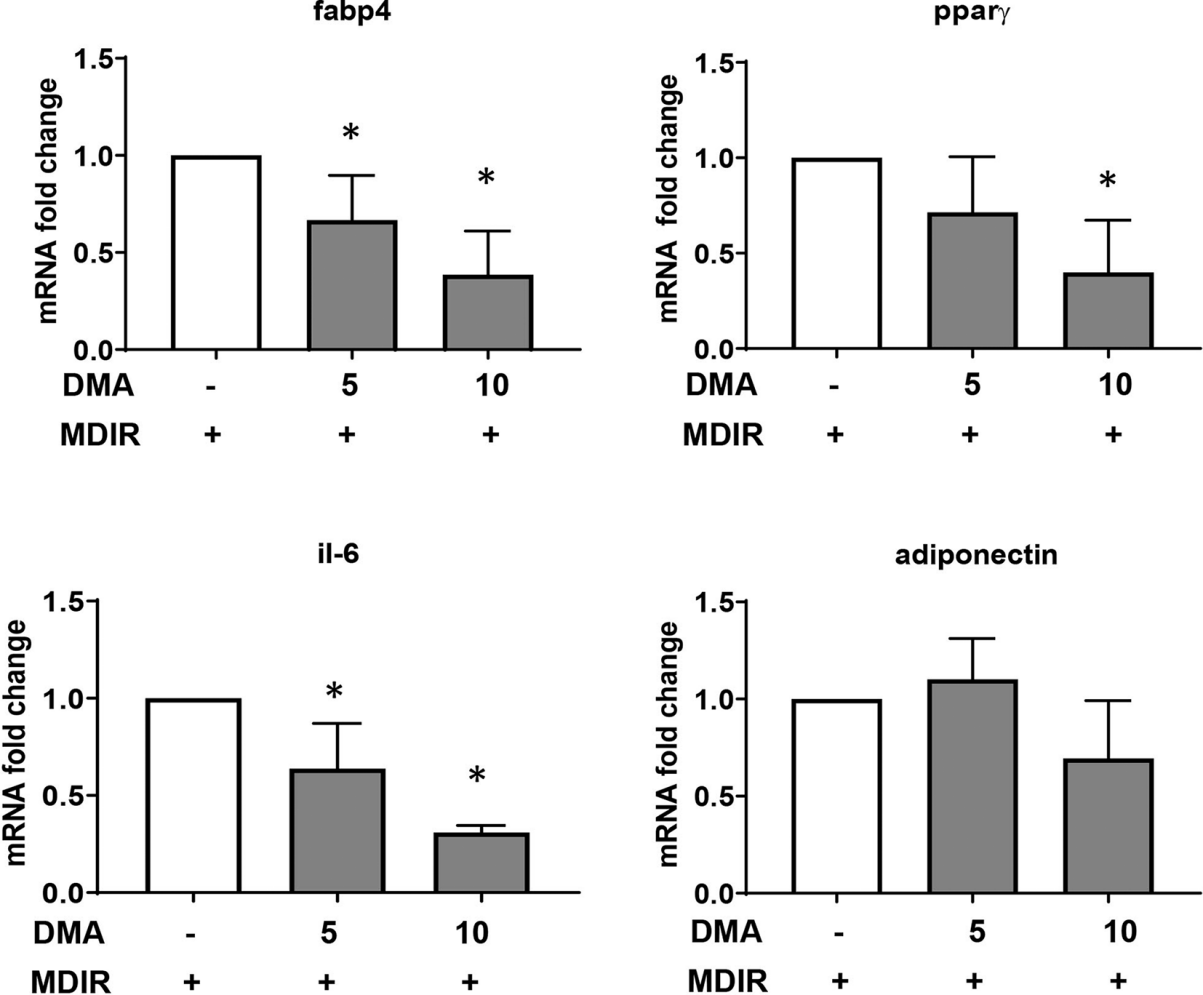
Gene expression of 3T3-L1 adipocytes. DMA (5 mM or 10 mM) was applied to 3T3-L1 cells throughout with the induction and differentiation media from day 0 to day 10. After day 10, RNA was extracted and gene expression of Fabp4, Pparγ, Il-6 and adiponectin was analyzed. RNA was extracted from 3 independent experiments. *p ≤ 0.05 vs. MDIR.

Differentiated cells expressed high levels of adipocyte marker, fatty acid binding protein 4 (Fabp4). In the presence of 5mM or 10mM DMA, the Fabp4 gene expression was reduced significantly in a concentration-dependent manner (**Figure 2**). The gene expression of PPARγ, a known marker expressed during early stages of adipocyte differentiation was significantly reduced by 10mM DMA. The inflammatory marker IL-6 was lowered by both 5mM and 10mM DMA. Adiponectin expression was not altered by DMA.

### DMA Inhibits Lipid Droplet Formation in C2C12 Cells

**Figure 3:**
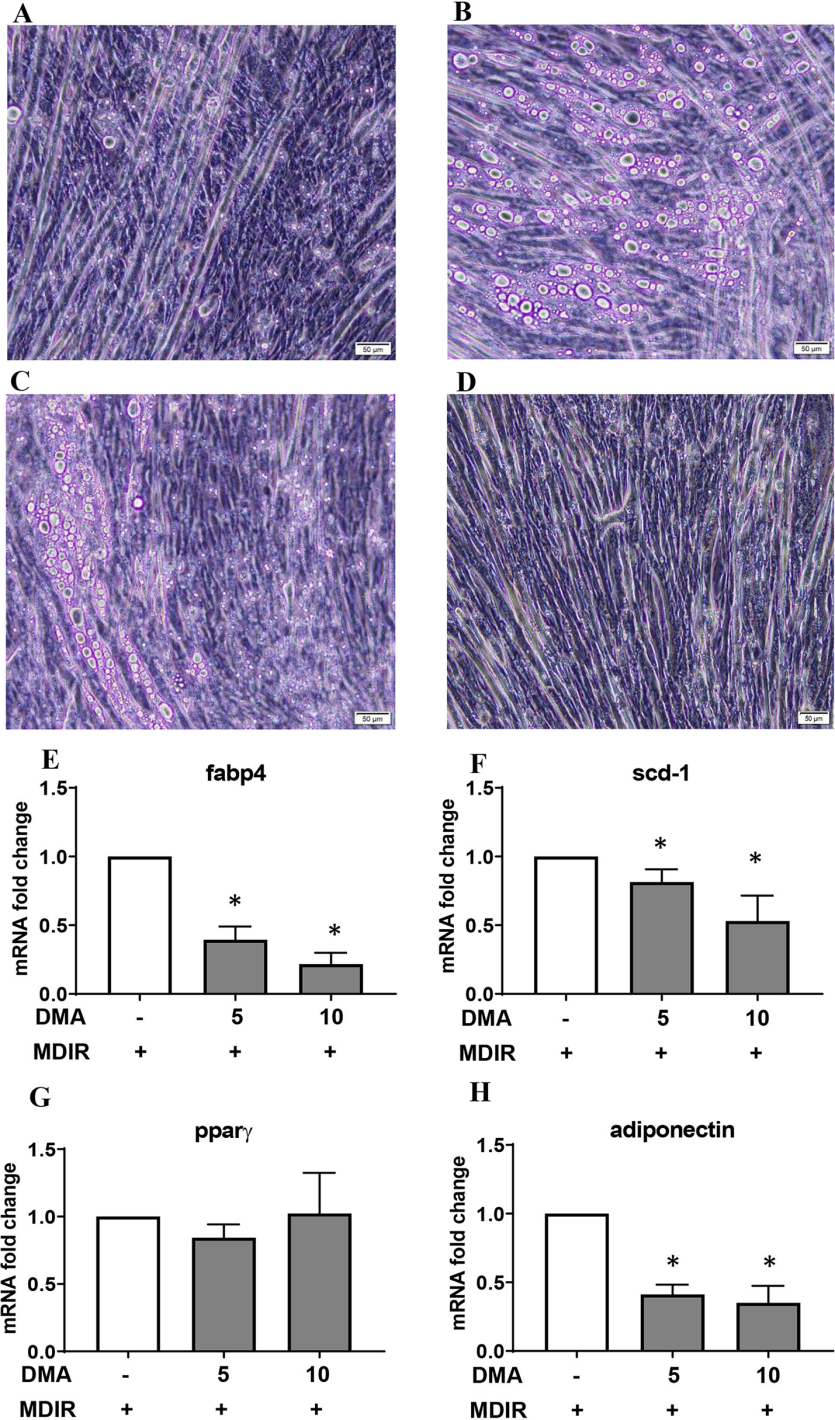
C2C12 cells were treated with MDIR in the absence or presence of DMA. The cells treated without MDIR differentiated to myotubes **(A)**. In the presence of MDIR, the cells developed lipid droplets but they still maintained the myotube characteristic **(B)**. With MDIR and 5mM DMA, the cells still had lipid droplets but in lesser proportion **(C)**. MDIR with 10 mM DMA did not have lipid droplets **(D)**. The photographs are representative of 3 independent experiments. Gene expression of C2C12 cells post-differentiation showed a decrease in expression of Fabp4 **(E)**, Scd-1 **(F)** and adiponectin **(H)** with 5mM and 10mM DMA. The expression of Pparγ **(G)** was unaltered. *p ≤ 0.05 vs. MDIR; n = 3.

Pluripotent C2C12 cells in the presence of 10% fetal calf serum (FCS) differentiate to myoblasts (**Figure 3A**). Treating the cells with adipocyte differentiation media pushed the cells towards oil droplet rich adipocytes (**Figure 3B**); however the cells maintained their myogenic characteristic (as seen by their ability to contract). Both 5 mM (**Figure 3C**) and 10 mM DMA (**Figure 3D**) were able to reduce the differentiation significantly.

Gene expression of Fabp4 and stearoyl-CoA desaturase (scd-1), a rate-limiting enzyme in the biosynthesis of fatty acids, was significantly reduced in the presence of DMA (**Figures 3E, F**). Interestingly, the expression of adiponectin was also reduced (**Figure 3H**) but the expression of PPARγ was a not affected (**Figure 3G**).

### DMA Prevents High-Fat Diet-Induced Increase in Body Weight

**Figure 4:**
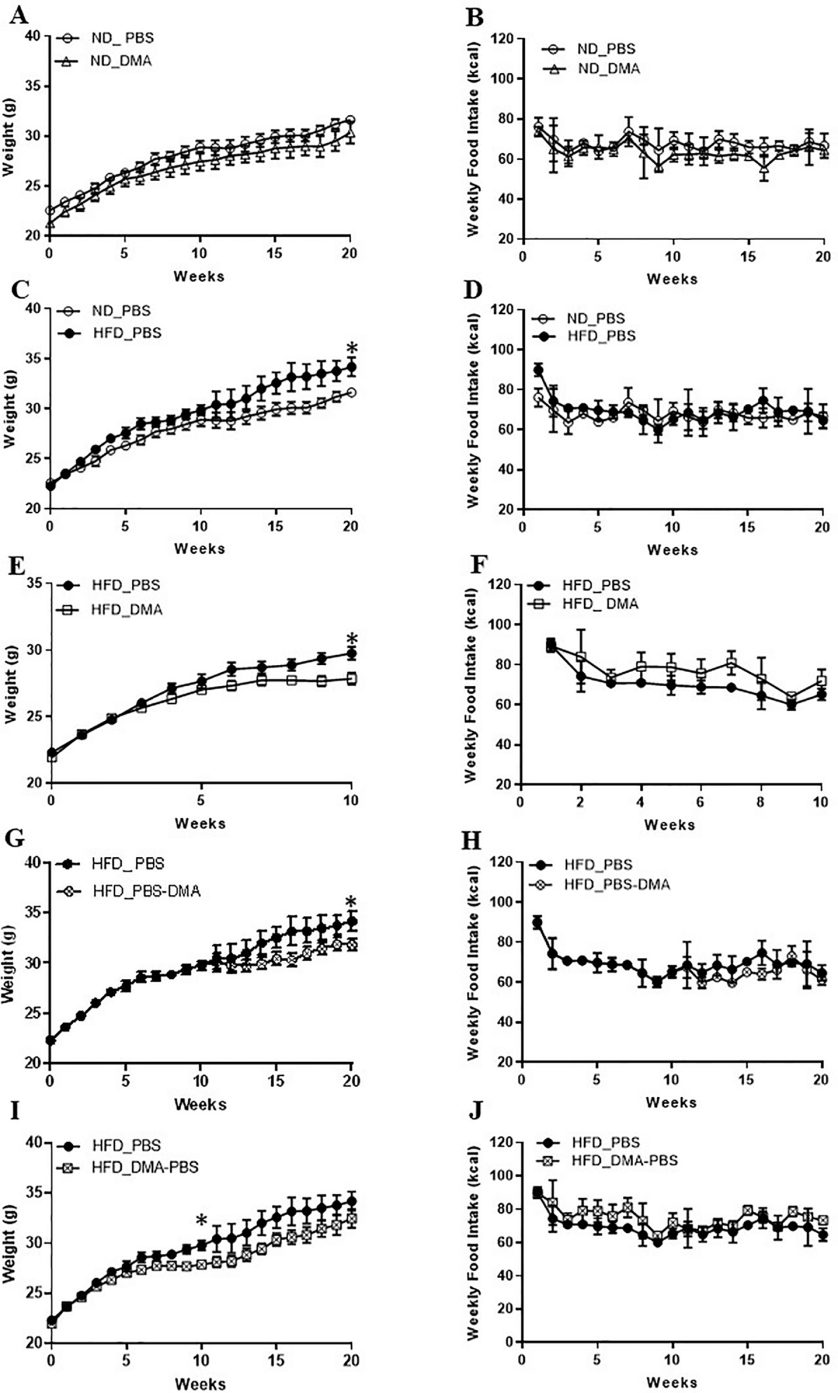
DMA prevented body weight gain in C57BL/6J mice fed with high-fat diet. Mice were fed with normal diet (ND) for 20 weeks and were injected once a week with either DMA or PBS. The body weight in grams **(A)** and the weekly food intake in kcal **(B)** was measured. On feeding the mice with HFD, the body weight was increased **(C)** however food intake (in kcal) remained similar **(D)**. Animals treated with DMA had lesser gain in body weight already after 10 weeks in the presence of HFD **(E)** and the food intake was similar between the groups **(F)**. When mice treated with PBS for 10 weeks were switched to DMA treatment for the next 10 weeks, the body weight increase was prevented even in the presence of HFD **(G)**. When mice treated with DMA for 10 weeks, were switched to PBS the body weight increased **(I)**. Food intake was similar across the groups **(H**–**J)**. *p ≤ 0.05, n = 5–6 mice/group.

Mice fed with standard chow and injected with DMA once a week gained body weight similar to the PBS treated animals (**Figure 4A**). Moreover, the weekly standard chow food intake (kcal) was similar between the mice treated with DMA or PBS (**Figure 4B**). Mice fed with high-fat diet (HFD) for 20 weeks had a significant higher body weight (**Figure 4C**) even when the weekly food intake (in kcal) was similar between HFD and standard chow groups (**Figure 4D**). When DMA was injected intraperitoneally once a week to HFD-fed mice, the body weight was significantly lowered by 10 weeks (**Figure 4E**). In HFD-fed mice when PBS was switched to DMA treatment at 10 weeks, the rise in body weight was controlled while in PBS treated mice the body weight kept increasing (**Figure 4G**). The lower body weight observed in the DMA-treated group in the presence of HFD until 10 weeks did not sustain when it was switched to PBS (**Figure 4I**). Interestingly, the weekly HFD food intake (kcal) was similar between the PBS and DMA treatment groups (**Figures 4F, H, J**), suggesting that the changes in body weight were independent of the calories consumed.

### DMA Reduced Adipocyte Hypertrophy

**Figure 5:**
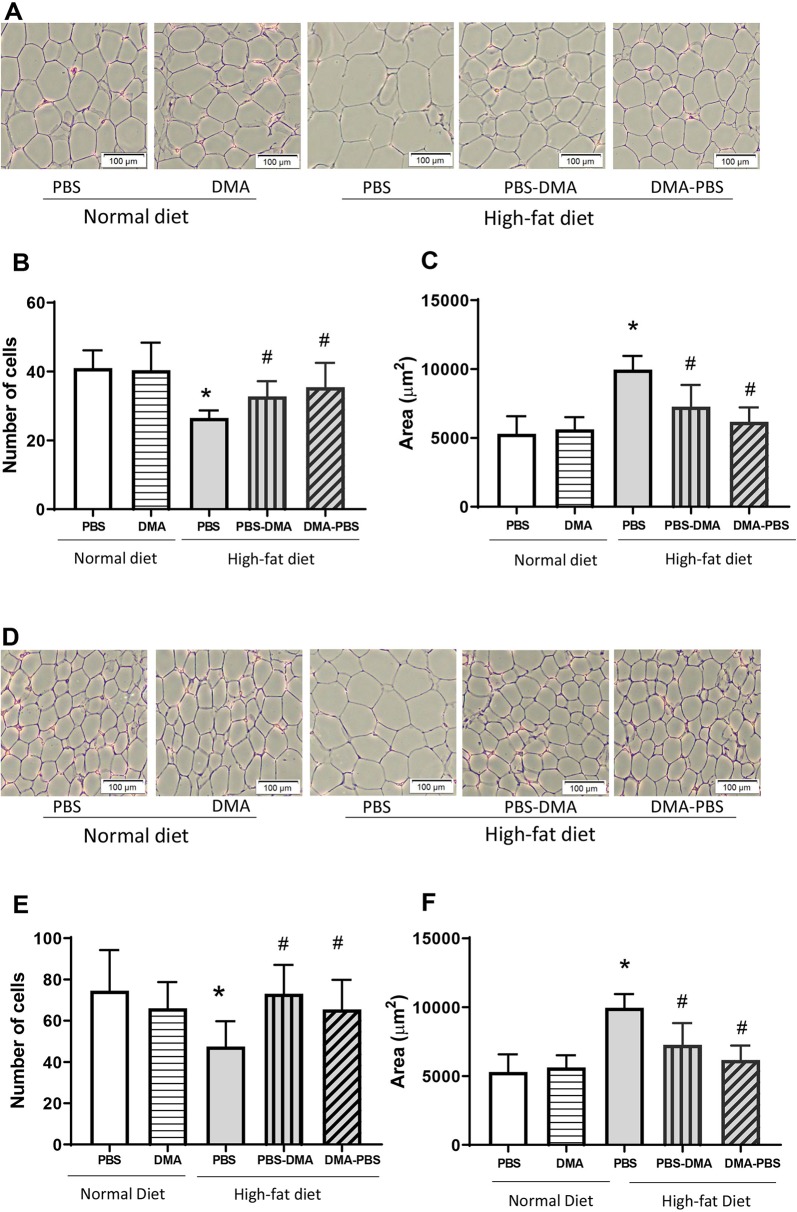
Increased adipocyte cell size was reduced with DMA treatment in epididymal **(A)** and subcutaneous adipose tissue **(D)**. Histological sections of epididymal and subcutaneous adipose tissue stained with H&E showed no difference in adipocyte cell number **(B**, **E)** and size **(C**, **F)** from mice fed normal diet and treated with either PBS or DMA. The epididymal and subcutaneous adipocyte area was significantly bigger from HFD fed mice **(C**, **F)** and the number of cells within a 100µm frame were quantitatively less **(B**, **E)**. Treatment with early DMA or late DMA reversed the increased adipocyte size with a parallel reduction in the adipocyte number. *p ≤ 0.05 vs. PBS-treated normal diet; ^#^p ≤ 0.05 vs. PBS-treated HFD. n = 5–6 mice/group.

Epididymal adipose tissue from mice fed with standard chow and treated with PBS or DMA had similar adipocyte number and size (**Figures 5A–C**). However, epididymal fat from HFD-fed mice had an increased adipocyte area and a lower adipocyte number compared with the epididymal fat from standard chow fed mice. Interestingly, epididymal fat from mice fed with HFD and either treated with PBS and then DMA or vice versa had a decreased adipocyte cell size and a concomitant increase in adipocyte number in both treatment groups. (**Figures 5A–C**). A similar trend was also seen in adipocytes from subcutaneous adipose tissue. The increased adipocyte size and decreased adipocyte number from subcutaneous fat of mice fed with HFD was reversed in the presence of early or late treatment with DMA (**Figures 5D–F**).

### DMA Reduces Fabp4 Gene Expression in Adipose Tissue

**Figure 6:**
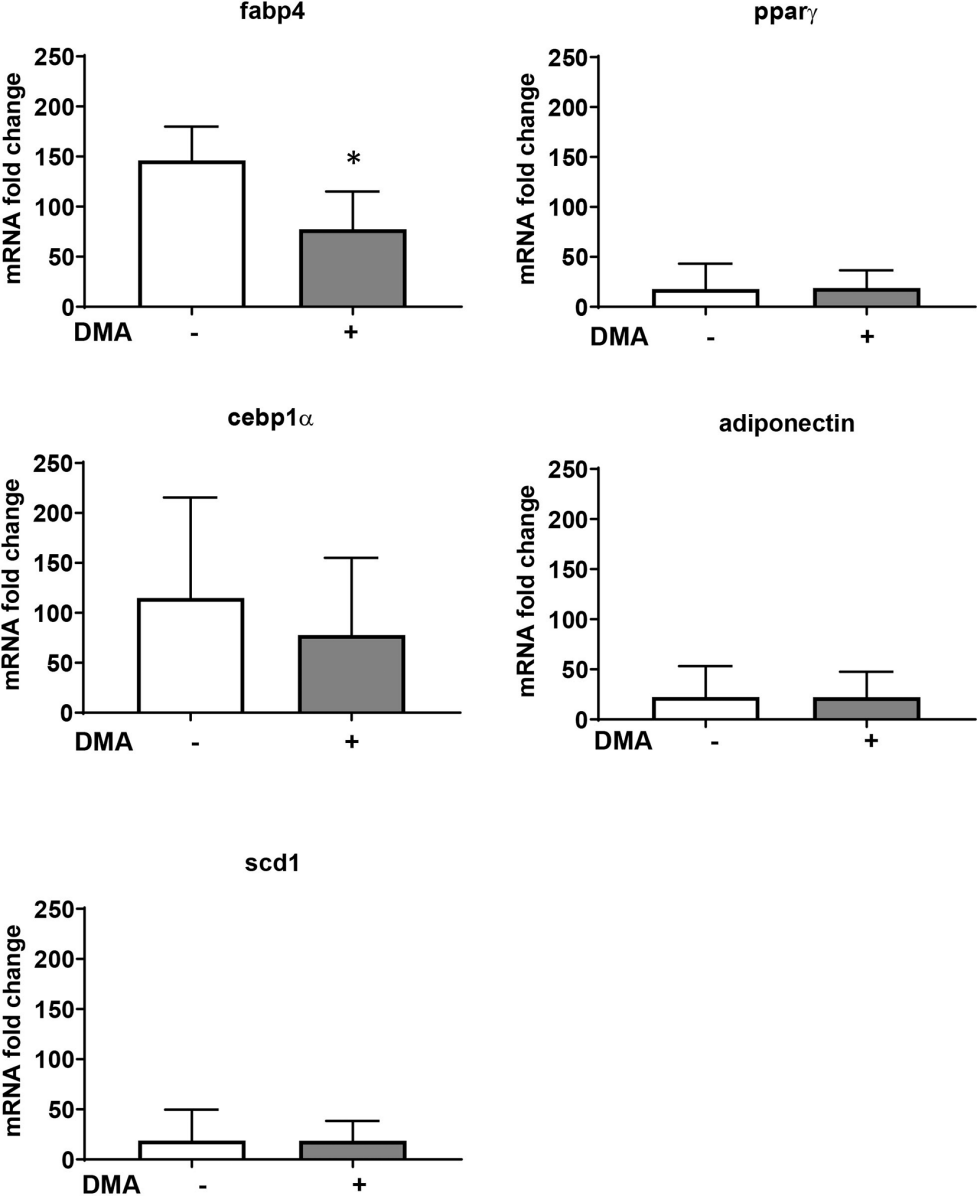
Gene expression from epididymal adipose tissue of mice fed with HFD and treated with either PBS or DMA. Only Fabp4 was significantly reduced in DMA treated mice. Other genes such as Pparγ, Cebpα, Scd-1 and adiponectin was not altered. *p ≤ 0.05 vs. PBS, n = 5 mice samples/group.

The increased expression of Fabp4 in the epididymal adipose tissue from mice fed with HFD was significantly lowered by treatment with DMA (**Figure 6**). Other adipocyte markers (Pparγ, Cebp1α, Scd-1, and adiponectin) were not altered in the epididymal fat by DMA treatment.

### Increased Blood Glucose Is Improved by Early DMA Treatment

**Figure 7:**
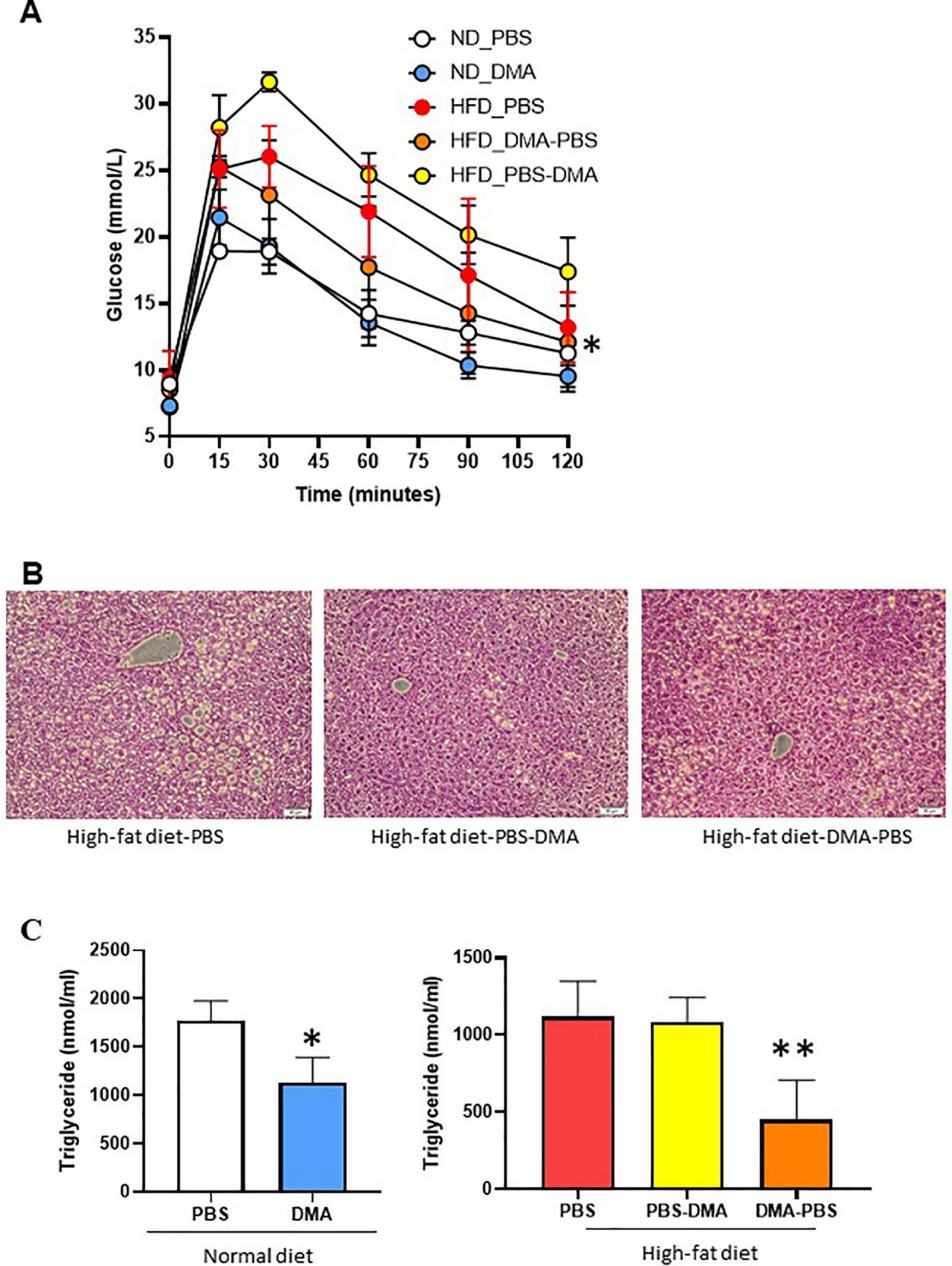
Glucose tolerance test to measure blood glucose levels (mmol/L) in mice fed with either normal diet or HFD and treated with PBS or DMA **(A)**. *p ≤ 0.05 vs. ND_PBS (n = 3 mice/group). Histological analysis with H&E staining indicated that the HFD fed mice had fatty liver **(B)**. There was a reduced fat cell formation in the mice fed with HFD and treated with DMA n = 5–6 mice samples/group. The plasma triglyceride levels were reduced in mice treated with DMA in the presence of normal diet **(C)**. *p ≤ 0.05 vs. ND_PBS (n = 3). The HFD-fed mice had reduced triglyceride levels only when DMA was treated early (C) **p ≤ 0.05 vs. HFD_PBS (n = 3).

Mice fed with HFD showed increased glucose levels as compared to normal diet fed mice. Interestingly, mice on HFD but treated with DMA from the beginning had an improved glucose clearance as compared to those treated with DMA after 10 weeks (**Figure 7A**).

### Increased Liver Adiposity

The liver samples from the mice fed with HFD and treated with PBS developed fatty liver. The HFD-fed mice treated with DMA indicated reduced deposition of lipid droplets (**Figure 7B**).

### Early DMA Treatment Results in Improved Triglyceride Levels

Mice on normal diet and treated with DMA for 20 weeks, had reduced triglyceride levels in plasma. Interestingly, the triglyceride levels in HFD mice were reduced only by early treatment of DMA (**Figure 7C**) and no effect was seen by late DMA treatment.

## Discussion

Tackling the increasing trends of obesity and the associated health issues such as type 2 diabetes, blood pressure and cancer is a major challenge. Many of these lifestyle-triggered conditions can be taken care of, controlled or prevented by altering and practicing lifestyle, which involves inculcating stress-free habits, eating healthy and moving regularly. However, the role of molecular intervention along with the positive lifestyle practices cannot be undermined.

In a previous work from our group using ovariectomized rats, we showed that the increased body weight induced by ovariectomy was reduced after treatment with DMA (Ghayor et al., 2017). In this present work, we have used diet-induced obesity as a more general and hormone independent overweight/obesity mouse model, which is a well-accepted model for studying obesity (Bhattacharya et al., 2008). In the present work, we dwelled deep to know if DMA intervened with the body weight increase induced by HFD feeding, a condition often associated with unhealthy lifestyle. We show that the small molecule DMA has the ability to intervene and control the increase in body weight induced by HFD (**Figure 4**), and reduce adipocyte hypertrophy (cell size). Moreover, the reduced adipocyte hypertrophy in both epididymal and subcutaneous adipose tissue was maintained even when the DMA treatment was discontinued. Thus, the findings suggest that DMA is a potential therapeutic candidate against HFD-induced adiposity.

DMA is a ligand for bromodomains and it is known to reduce LPS-induced expression of inflammatory cytokines in cell lines (Ghayor et al., 2017; Gorasiya et al., 2018; Pekson et al., 2016). Obesity is a low grade chronic inflammation and amongst the various inflammatory molecules, the levels of IL-6 is increased in the serum of obese patients (Roytblat et al., 2000). Our results with 3T3-L1 adipocytes show that the basal expression of inflammatory molecule IL-6 was lowered by DMA in a concentration-dependent manner (**Figure 2**).

Increased inflammation is often associated with the expression of fatty acid binding protein (Fabp4), a molecule that is significantly elevated in morbid obese individuals. Fabp is a small cytoplasmic lipid binding protein and is known to mediate fatty acid transportation, trafficking and metabolism. Increased expression of FABP has been shown to mediate age-related inflammation and metabolic diseases (Charles et al., 2017). The plasma FABP4 levels have been reported to be upregulated in obese patients and positively correlate with BMI and insulin resistance (Terra et al., 2011). In our study, the increased expression of Fabp4 (also known as alipoprotein2), was reduced consistently by DMA in epididymal adipose tissue, 3T3-L1 and C2C12 cells. Calorie-restriction which is opposite to *ad libitum* high-fat diet feeding improves overall metabolic health in mice. Interestingly, calorie restriction and Fabp deficiency share many common molecular features (Charles et al., 2017). Since the elevated levels of Fabps have been implicated in obesity, diabetes and atherosclerosis therefore molecular inhibitors for Fabps are of therapeutic interest (Furuhashi et al., 2007; Floresta et al., 2017). And indeed small molecule inhibitor for FABP4 have shown to also reduce inflammation (Gong et al., 2018). In this regard, reduced Fabp4 expression by DMA is of importance and can be explored to treat diseases where FABP4 expression is upregulated.

PPARγ is a master regulator of adipogenesis and aids in the induction and formation of mature adipocytes. It has been suggested that the expression of PPARγ is not extremely critical in maintaining mature adipocytes (Moseti et al., 2016). On the contrary, FABP4 expression is more stable in mature adipocytes. Additionally, in adipose tissues high expression levels of FABP4 reduces the expression of PPARγ (Garin-Shkolnik et al., 2014). These may be some factors on why we could not detect any change in PPARγ gene expression in the presence of DMA, Moreover, the other added reasons may be:

1. Type of cells- The reduction in PPARγ expression by DMA was only evident in adipocytes (3T3-L1) and not in pluoripotent cells (C2C12) and in adipose tissue, which is a mix of many cells type.
2. Sensitivity- There may only be a small reduction in the expression of PPARγ by DMA and this was only detected in adipocyte specific 3T3-L1 cell line.

We did see a consistent decrease in FABP4 expression in the presence of DMA in all the three models investigated. PPARγ is known to regulate the expression of FABP4, however other pathways such as inflammation also regulate FABP4 expression. Work from our group (Ghayor et al., 2017), has shown that DMA reduces the inflammation and probably this reduction in inflammation has a contribution in the reduction of FABP4 expression.

Adiponectin is not only expressed in adipocytes but it is also known to be expressed in soleus muscles and in C2C12 myoblasts (Goto et al., 2013). In our study, C2C12 cells when treated with adipogenic media took up the lipid droplets but maintained muscle contractile characteristic. Adiponectin is generally accepted as an anti-inflammatory molecule and its levels are reduced with increased body weight in both animal models and in humans (Ouchi and Walsh 2007; Nigro et al., 2014). However, in this work, treatment with DMA lowered adiponectin levels in C2C12 myotubes, without affecting its expression in 3T3-L1 adipocytes and epididymal adipose tissue. More recently, the term adiponectin paradox came to the fore, which suggests that cardioprotective effect of adiponectin is also associated with adiponectin-dependent increase in advanced cardiovascular disease pathogenesis (Woodward et al., 2017). In skeletal muscles and in C2C12 myotubes, inflammatory cytokines has been reported to increase the levels of adiponectin *via* inducible nitric oxide synthase (iNOS) (Delaigle et al., 2004). This suggests that adiponectin possibly plays an inflammatory role depending on the cellular context. Thus the lowering of adiponectin expression by DMA in C2C12 fatty myotubes, in this study, may be related to the possible inflammatory effect that adiponectin influences in these myotubes.

The findings with 3T3-L1 cells indicate that the long-term treatment with DMA not only inhibits the differentiation of adipocytes but also reduce the matured adipocytes, as indicated by reduction in Oil red O staining. Indeed, only one-time application of DMA even at high concentration (10 mM) did not inhibit adipogenesis in 3T3-L1 cells. Our results with cell culture showing that the continuous DMA treatment has an effective anti-adipogenic effect was also reflective in the mouse experiments. When DMA treatment was switched to PBS, the body weight of the animals that was under check by DMA, under HFD feeding, was altered thus indicating that continuous DMA treatment is essential to prevent weight gain. In summary, the *in vitro* and *in vivo* results suggest that DMA application has to be sustained to obtain a strong and effective anti-adipogenic response and to reduce adiposity even in the presence of HFD.

DMA is FDA approved drug carrier solvent and has been reported to have no toxic effect in humans. Pediatric patients with myeloid leukemia when given DMA intravenously to deliver busulfan reported no accumulation of DMA in the body (Hempel et al., 2007). Moreover, the dose of DMA applied was in the range of 70 mM, which was far higher than what we have used in our study. In the present work, the dose for DMA was based on our earlier work with rats (Ghayor et al., 2017) from our group and initially chosen based on the work by Bartsch et al. (1976). The authors suggest that in mice DMA administration *via* i.p. route should not be more than 1/4^th^ the LD_50_ for DMA. Moreover, the concentrations used for DMA in the cell lines was based on an earlier work from our group with NMP (Gjoksi et al., 2016)

Our findings indicate that the metabolic parameters such as blood glucose levels and triglyceride levels were improved only when DMA was given early to the animals in the presence of HFD. This suggests that once the insulin resistant state or high triglyceride levels are established by HFD feeding, it is then difficult for DMA to revert it back to the normal. However, further work and other metabolic parameters need to be looked at to get a more conclusive answer.

The animals used in the study were 26 weeks old (at the end of the experiment), thus studies with older mice fed with HFD diet will provide us clearer information on the effect of DMA on adulthood obesity. These studies will also provide us information if DMA affects longevity. Since DMA was able to significantly reduce Fabp expression, this study provides hints to use DMA in other disease models like atherosclerosis where Fabp expression is enhanced (Tu et al., 2017).

In conclusion, the findings of this study suggest that DMA has a strong potential to control adiposity and prevent the fat-related increase in body weight contributed by HFD feeding. This would contribute in preventing overweight/obesity in the presence of unhealthy food intake. Hence, we propose DMA as a potential therapeutic candidate against weight gain/obesity and diseases associated with it based on the available data.

## Data Availability Statement

All datasets generated for this study are included in the article/supplementary material.

## Author Contributions

IB, CG, and FW designed experiments. IB, AP, and CG performed the experiments. IB analyzed the data. IB and FW wrote the manuscript.

## Funding

The research work was supported by grants from Swiss National Science Foundation to FW.

## Conflict of Interest

The authors declare that the research was conducted in the absence of any commercial or financial relationships that could be construed as a potential conflict of interest.
